# Supplementary material for: Effectiveness of BNT162b2 and CoronaVac in children and adolescents against SARS-CoV-2 infection during Omicron BA.2 wave in Hong Kong
Source: Commun Med (Lond). 2023 Jan 5;3:3. doi: 10.1038/s43856-022-00233-1 (PMC9813885; doi:10.1038/s43856-022-00233-1)
Supplement: Supplementary file 2 — Description of Additional Supplementary Files [file 43856_2022_233_MOESM2_ESM.pdf]

Description of Additional Supplementary Files

File Name: Supplementary Data 1

Description: The aggregate dataset for all Tables and Figures
